# Opting in to paid work in old age

**DOI:** 10.1093/geront/gnag196

**Published:** 2026-08-12

**Authors:** Loretta G Platts, Kevin E Cahill

**Affiliations:** Department of Psychology, Stockholm University, Stockholm, Sweden; Center for Advanced Study in the Behavioral Sciences, Stanford University, Stanford, California, United States; Center on Aging & Work, Boston College, Chestnut Hill, Massachusetts, United States

**Keywords:** Post-retirement work, Third age, Bridge employment, Encore career, Social inequality

## Abstract

Older people’s paid work takes on a distinctive character once retirement on an adequate pension has become a realistic alternative. Rather than retiring, these older workers are opting in to the labor market by continuing their preexisting work arrangements, adapting them, or seeking new jobs. This way of working—facilitated by old-age income security systems—merits both theoretical and empirical attention. The current paper offers a multidisciplinary account of the distinctive nature of these opt-in jobs and suggests potential conditions and mechanisms that underpin them. We make the case that these workers are no longer compelled to work for pay by financial necessity, as retirement income systems have removed or substantially eased breadwinning pressures, and that they are defying social conventions calling on them to retire. These opt-in older workers typically seek to maximize the intrinsic rewards of paid work while defending their hard-won freedom over their time and energy. Two mechanisms appear to generate the distinctive nature of these jobs: differential selection of older adults out of the labor market and changes in the nature of jobs held by people working in the years normally set aside for retirement. We outline the value of opt-in workers for developing gerontological theory and suggest several promising avenues for empirical research, including the influence of retirement income systems upon working lives, strategic interactions between employees and employers, and the changing priorities of older people themselves as they age.

“I mean, it is naturally different, if you must [work], if there is a must behind it. Well, then I can imagine that it’s a different situation then. But I don’t have to, I can. And that’s something different.” Mrs L., 69 years, German saleswoman (Wagner & Wachtler, 1996, p. 15, authors’ translation)

“I steer over my working time now. The disadvantage is that you have got older, one should have been able to do this at 40. To steer over one’s own time, it’s worth gold.” Hulda, 68 years, Swedish midwife (Platts et al., 2023a, p. 1322).

In the quotes above, Mrs L. and Hulda, interviewed almost thirty years apart in Germany and Sweden, described their experiences of working at a time of life when paid work had become voluntary for them. Both women received old-age pensions and belonged to an age-group in which retirement from work is a conventional lifestyle. Paid work had shifted from a “must” to an activity that they opted in to.

This ability of some older workers to opt in to the labor market at older ages is a product of what the American historian William Graebner called the “triumph of retirement” (1980, p. 241). Retirement was invented in part to let governments and businesses ease older people out of the workforce. In many high-income countries over the twentieth century, a plethora of new social protection arrangements—age-graded public pensions and social security, occupational and private pensions, and accompanying provisions such as health insurance—gradually created a fixed retirement age and transformed the economic realities of aging (Graebner, 1980, pp. 215–225; Kohli, 1987; Thane, 2000, p. 255). Accompanied by gains in average longevity and healthy life expectancy, these policies created a norm of full-time retirement at ages when many people still felt reasonably fit (Harper & Thane, 1989). Some decades later, governments, eying the costs of their public pension commitments, sought an about-turn. Social security reforms were implemented in many high-income countries to strengthen financial incentives to work at older ages, delaying departures from workforces (OECD, 2025, p. 203) and increasing rates of paid working even after pensionable age (Börsch-Supan & Coile, 2018). Some in these age-groups work in response to financial pressures (Di Gessa et al., 2018; Flynn, 2010), much as most older people did in the past, and excellent research has scrutinized the situations of these workers (Lain et al., 2020; Lain & Phillipson, 2019; Yang, 2011). Others, who can afford to retire, form an emergent group that is opting to work for pay in those years that are usually set aside for retirement.

Research in a range of fields offers insights into post-pensionable-age or post-retirement workers’ situations and experiences, but the picture provided is a partial one. Various attempts have been made to classify jobs located in the work–retirement gray zone, leading to a proliferation of inconsistently defined and a-theoretical concepts (Lain et al., 2022), such as retirement job, semi-retirement or partial retirement. Difficulties classifying jobs are generated by the manifold ways in which older people work for pay, including variations in the worker’s own self-perceptions, working hours, labor market histories, whether they receive pensions and what types, and their age in relation to statutory retirement age (Beehr & Bennett, 2015; Leinonen et al., 2022). Taking a longitudinal perspective, these jobs can be viewed as part of an extended retirement process, in which bridge jobs can be defined as temporary or part-time spells in new jobs following career employment, and phased retirement as reductions to working hours in the career job (Cahill et al., 2015). A complementary perspective is provided by the career development literature, which spotlights how some workers in these age-groups shift toward more meaningful work in renewed or encore careers (Freedman, 2017; Power, 2009).

A vital complement to these approaches is a life course framing that portrays retirement both as a social institution, managed by laws and policies, and as a cultural ideal for later life (Cain, 1974; Kohli, 1986). In North American and many European countries, retirement is “adulthood’s great project of deferred gratification” (Ekerdt, 2004, p. 4), the freedom at last to devote oneself to leisure and other non-market activities (Anderson & Gettings, 2020; Wainwright et al., 2019), and a “well-earned reward for a lifetime of paid work” (Moen & Chermack, 2005, p. 100). The chronological age at which it is appropriate to claim that reward and retire is influenced by norms, laws, and policies regulating retirement and associated income systems (Riekhoff, 2024; Settersten & Mayer, 1997). These institutions are in flux: many high-income countries have in recent decades sought to retain older workers in the workforce with policies that eliminate or scale back mandatory retirement ages, special pre-retirement schemes, and restrictions on combining work and pensions (Börsch-Supan & Coile, 2018; Kuitto & Helmdag, 2021). Further, defined benefit schemes, providing lifelong predictable pensions, have been declining in prominence in favor of arrangements that shift longevity and financial market risks to individuals (e.g., defined contribution schemes, voluntary pension schemes, private savings, and earnings) (Hinrichs, 2021; Quinn & Cahill, 2016). Facing increased risks, responsibilities, and choices, growing numbers of older people are remaining in the workforce in the retirement phase of the life course—as we explain below, often by choice rather than financial necessity—navigating their working lives “in the absence of standardized blueprints” (Moen & Oh, 2026).

In this reading of the life course scholarship, destandardization is partial (cf. Guillemard & Rein, 1993); working in the life course phase of retirement, although increasingly common, remains non-normative, and is unreinforced by mindsets and institutions. Resources also matter—where social protection in old age is sufficiently generous to decommodify these workers from the labor market, it removes the economic compulsion to work (cf. Platts et al., 2023a; Scherger, 2015; Wainwright et al., 2019). These two aspects operate in concert: upon reaching the retirement years, both financial and normative pressures to remain in the workforce ease. This lack of duress is exemplified by Mrs L.’s and Hulda’s accounts of the freedom inherent to their working arrangements. The result is that *these “opt-in” older workers have chosen employment when full retirement is a realistic option for them, and they are in work during the years conventionally set aside for retirement*. Taking the argument one step further, we will make the case that the job needs to be sufficiently satisfying to contribute to a more agreeable lifestyle overall than full retirement would offer; otherwise the person would retire. And we claim that having retirement as a fallback can offer these workers leverage vis-à-vis employers or clients that lets them adjust their jobs to match their specific requirements at this stage of life.

In what follows, we provide a comprehensive account of the distinctive character of what we call “opt-in” employment, by assembling fragments of knowledge that are dispersed through the fields of gerontology, economics, psychology, sociology, and anthropology. Drawing these ideas together lets us specify the testable conditions and mechanisms that underpin the distinctive nature of the jobs held by opt-in older workers. Hence, the main contribution of this paper is in providing a solid platform for future research. To this end, we outline the interest of these workers for developing gerontological theory and lay out promising avenues for empirical studies.

## A brief overview of opt-in employment in later life

Over the last four decades, a small, scattered, multinational, and multilingual literature has shed light on the working lives of opt-in older workers. The fullest accounts of these workers’ situations stem from Germany (Fréter, 1993; Hokema, 2016, 2018; Wachtler & Wagner, 1997; Wagner & Wachtler, 1996), Hungary (Szalai, 1991), Sweden (Bengs & Stattin, 2018; Platts et al., 2023a), the United Kingdom (Barnes et al., 2004; Hokema, 2016, 2018), and the United States of America (Lynch, 2012; Meltzer, 1981), as well as international comparisons of Japan, Sweden and the United States (Platts et al., 2023b) and Germany and the United Kingdom (Hokema, 2016, among other papers from this research group). The earliest accounts do not cross-reference each other, making their similarities all the more striking and strengthening the case that opt-in employment has a distinctive nature.

We introduce the fundamental dynamics of opt-in employment through Júlia Szalai’s (1991) adroit analysis of industrial workers in communist Hungary. Jobs in the formal economy were poorly paid, dangerous, and onerous, imposing rigid working hours and compulsory overtime. But pensioners were no longer obliged to have a formal job. Instead, they could devote themselves to agriculture on their small family plots, a less constraining form of employment that let pensioners supply their household with food and sell any surplus for profit. Around one-quarter of male pensioners engaged in a second form of working: being enticed back to work for their former firms with offers of more flexible hours and no more compulsory overtime. Szalai observed how these more favorable working arrangements resulted from retired workers’ improved bargaining position vis-à-vis their former employers (1991, pp. 350, 352). We note in passing that Szalai’s account is set in an austere communist context, which suggests that these jobs may be more widespread than one might initially presume.

## What do opt-in jobs look like?

Opt-in jobs look alike in several ways: (1) Financial motives for working still matter, but have largely given way to intrinsic motivations as part of a broader shift in priorities; (2) idiosyncratic and, at first glance, paradoxical changes in job quality have taken place; (3) the freedom that retirement promises is not endangered; and (4) work arrangements are often individually negotiated, with an eye to near-term costs and benefits.

Without the same pressure to secure a livelihood in the labor market anymore, earnings stop being the primary motivator for working (Fréter, 1993; Hokema, 2016, p. 227; Lynch, 2012, p. 119; Platts et al., 2023a; Reynolds et al., 2012; Wachtler & Wagner, 1997, pp. 85, 98). Opt-in older workers still value their paychecks, because they provide economic resources and signal that these workers still matter (Fréter, 1993, p. 91; Lynch, 2012, pp. 10, 115). But pay is less important than before: a large American survey of working retirees found that they had accepted a 57-percent fall in hourly wage in their new job and yet still preferred their new job to their old one (Johnson et al., 2009, p. 18). Opt-in older workers also deprioritize health insurance, career perspectives, status, and employment security (Fréter, 1993; Hokema, 2016, pp. 231–232; Johnson et al., 2009, p. xi; Lynch, 2012, p. 110; Wachtler & Wagner, 1997, p. 98). The priority now is the immediate intrinsic rewards of working: meaning, belonging, recognition, connection, contribution, and competence (Wachtler & Wagner, 1997, pp. 85–91), and not least the fun and enjoyment that paid work can provide (Hokema, 2016, p. 227; Platts et al., 2023a).

Post-pensionable-age workers tend to report higher job satisfaction and more favorable physical and psychosocial working conditions than younger workers (Platts et al., 2023b). In a large European survey, workers over pension age reported lower exposures to tight deadlines, various dangers, and adverse social behavior, and were less likely to view their jobs as damaging their health (European Foundation for the Improvement of Living and Working Conditions, 2017). While these are all self-reports, and may reflect the joys simply of being in work (Jahoda et al., 2002), it is difficult to imagine that they do not coincide with changing external realities. These reports of good working conditions occur despite the frequent occupational downgrading that takes place (Hokema, 2016, p. 196), such as the pensioner museum attendants in Fréter’s (1993) study who had switched into a less skilled, lower-paid job that involved weekend working and seemingly monotonous hours of standing to monitor the galleries. The resolution to this apparent paradox is that these jobs fit the workers’ changed priorities in retirement, such as avoiding stressful demands that might exacerbate their health problems (Fréter, 1993, pp. 85, 88).

To the extent that older people have non-market activities they wish to do, the jobs that they are opting in to must not endanger the freedom that retirement offers: time and energy should be preserved for other valued activities (Deller & Maxin, 2008; Meltzer, 1981; Wagner & Wachtler, 1996). In order to contain their work commitments, opt-in older workers find nonstandard contracts, accepting or even appreciating irregular working hours and attendant fluctuations in earnings (Platts et al., 2023a; Wainwright et al., 2019). Many are in “not-so-big jobs,” in which they have reduced their physical presence at work and made gains in schedule control (Kooij et al., 2015; Moen, 2007; Szalai, 1991), leading, for some, to a near-paradisiacal work-life balance (Lynch, 2012, p. 106).

For people in employment at ages that are conventionally viewed as the retirement years, this phase of working life becomes an improvisational, “do-it-yourself” project (Cahill et al., 2011; Moen, 2016a). Working arrangements are often individually negotiated and personalized (Platts et al., 2023a; Wagner & Wachtler, 1996), whether through quietly customizing the job (job crafting, Wrzesniewski & Dutton, 2001) or negotiating with the employer for a personalized, nonstandard arrangement (idiosyncratic deals, Rousseau, 2015). Typically, opt-in older workers weigh up reasonably immediate costs and benefits, confining planning to the short-term or resisting planning entirely (Barnes et al., 2004; Fréter, 1993; Hokema, 2016, pp. 197–199), since any future plans are viewed as vulnerable to disruption by devastating illness or death (Carstensen, 2021; Platts et al., 2023a).

## What are the predisposing conditions, mechanisms and boundary conditions that underlie opt-in employment for older workers?

**Figure 1:**
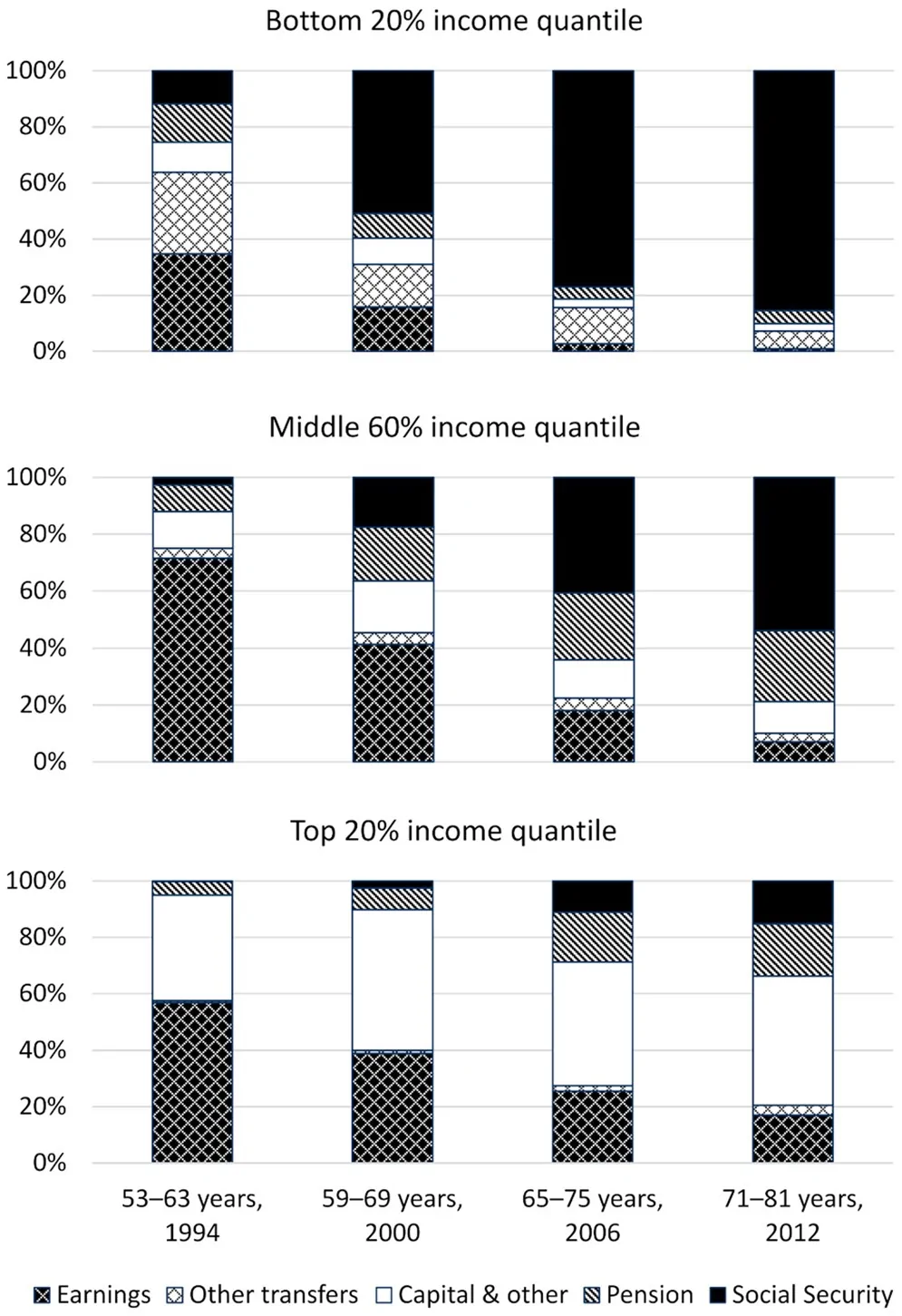
Changing income sources for cohorts of low, middle, and high earners in the United States. Created by the authors from Hungerford (2020, Table 3).

Obviously enough, what distinguishes opt-in older workers is that they no longer *have to work*, but *they can*. The first of these two predisposing conditions is that these workers no longer *have* to be in the labor market because they have aged into the life course phase of retirement. The retirement mystique, which emphasizes leisure in old age, is underpinned by the commencement of pensions and other parts of retirement income systems (Moen & Roehling, 2005, p. 143). While countries differ greatly in their institutional arrangements, and individuals’ working lives are also highly variable, the overall pattern in most high-income countries is still one of strong institutional influence over the life course, as age-graded public and occupational transfers dominate the pension mix in many rich countries (Ekerdt, 2004; OECD, 2025, p. 209; Scherger, 2024). Attempts by Western European governments to lengthen working lives have led to increases in preferred retirement ages (Hess, 2017), but retirement as an ideal and the practice of retiring within a relatively narrow band of ages has largely held, with the norm being retirement around pensionable ages in Western Europe (Börsch-Supan et al., 2022, Figures 3.10 and 3.11) and the United States (Truesdale et al., 2022, Figure 1.1). In the United States, for example, older adults’ sources of income shift as they age—from income sources that are earned toward those that are paid simply because one is still alive (Figure 1). Social protection eases breadwinning pressures most when it is universal, permanent, paid reliably, and protective of living standards (Esping-Andersen, 1990, p. 47). The prototypical opt-in older worker has been completely or near-completely decommodified from the labor market, and economic survival no longer motivates work. However, even less protective income security systems, which merely ease breadwinning pressures, may enhance a person’s room for maneuver in the labor market.

The second predisposing condition is that the potential opt-in worker *can* remain in their job or find a new one. This requires that the person is willing and able to work (supply side) *and* that a suitable job is available at an acceptable wage (demand side). On the supply side, older people’s willingness and ability to work for pay are affected by a vast range of factors comprehensively reviewed by Fisher et al. (2016). It is helpful to take a cost-benefit perspective in which a person will be willing to take a job if they judge that the job’s net benefits, broadly speaking, exceed those of the alternative option of full retirement. Social norms also matter: a person who views paid work at older ages as a commonplace, legitimate, and valued activity will be more likely to do it. Aside from a person’s willingness to work, many factors affect a person’s capacity to work for pay, including their human capital (health, qualifications) and their other competing responsibilities, such as grandparenting (De Preter et al., 2013). On the demand side, sectoral variation can be substantial (Lain et al., 2026). Desirable jobs are easiest to find and keep in tight labor markets offering plentiful freelancing, flexible, or part-time job opportunities, and where workplaces are age inclusive and prohibit age discrimination.

We suggest that this pair of conditions sets into motion two mechanisms that shape the nature of opt-in employment. The first mechanism is *selection*, and its logic is as follows: If most people retire when pensions and accompanying age-related provisions offer income replacement, only a minority remain in the workforce, strengthening selection processes that determine *which* workers hold jobs after pensionable age. Older adults will be more willing to remain in the workforce if they enjoy working, are in better situations at work, or see possibilities to improve their employment conditions, all else being equal. But all else is not equal, and it is more advantaged workers who will be more likely to stay in the workforce. Certain advantages, such as being in good health at older ages or possessing qualifications that are valued in the labor market, are influenced by inequalities at the intersections of characteristics such as gender, age, race, and class (Moen & Oh, 2026).

There is less evidence for a second mechanism—*changes* for the better in job quality—but we believe it is intriguing and merits study. Following Szalai (1991), we argue that, once armed with secure pension income or another old-age income source, potential opt-in workers have the credible alternative of stopping working completely, or stopping temporarily to search for a more attractive job. These fallback options enhance their leverage vis-à-vis employers, and employers will need to provide sufficiently enticing offers if they wish to employ opt-in older workers (Hayward et al., 1994; Kohli & Rein, 1991, p. 18). This mechanism helps make sense of recent evidence from Sweden demonstrating that post-pensionable-age workers are employing strategies such as reducing their working hours or switching into self-employment to direct their jobs toward preferred tasks and relationships (Platts et al., 2023a; Sacco et al., 2022).

The potential boundary conditions of this form of working relate to absences of the conditions that allow older people to freely opt in to work. Globally, pension and social insurance systems decommodify only a minority of older people from the labor market (Pallares-Miralles et al., 2012, p. 75). Even countries with comprehensive retirement income systems do not provide everyone with adequate incomes in retirement; poverty alleviation and wage replacement by old-age pensions assume decades of working in jobs with pension coverage (Hinrichs, 2021; Lain et al., 2019). Moen (2016b, p. 251) noted how life course institutional clocks that underpin retirement income security are based on “a white, middle-class and unionized working-class male template of work.” Disadvantaged groups, such as women, immigrants, people racialized as non-white, and the ununionized working-class, who are vulnerable to having low-paid jobs and interrupted careers, are less well served by pension and social insurance systems that are based on a lifetime of employment. Reflecting accumulating inequality over the life course, people who have led disadvantaged lives have higher odds of facing income shortfalls later in life while being able to do little about it, due to difficulties finding decent work in old age (Moen et al., 2022). In short, the financial and hedonic benefits that opt-in older workers experience are likely to be directed predominantly to those who were already in good situations, reinforcing life course patterns of disadvantage and exacerbating financial inequalities.

## Future directions

Study of opt-in older workers offers promising terrain for developing gerontological theory. Instead of retiring on time, per pension eligibility ages, these workers are finding jobs that are often more attractive than conventional perspectives on older workers might lead researchers to expect. These jobs are rooted in twentieth-century arrangements for pensions and retirement, and have flourished as the work-retirement lockstep loosened. They connect central theoretical concerns that extend into the fields of gerontology, sociology, organizational and management studies, social policy, and economics: age norms, social protection, individual agency, practices of working and retirement, and social inequality, as well as the interactions between them, such as the moderation of economic risks at older ages by social insurance and pension systems. These jobs center issues of power and empowerment that are less explored, but just as pressing.

Empirically, there is much to learn about these opt-in jobs, starting with where they are found and how widespread they are. What fraction of workers over conventional pension, social security, and retirement ages freely opt in to the workforce? Research could examine how the quantity of these jobs is influenced by macro-level factors including economic development, welfare regime, labor market characteristics, cultural expectations, retirement rules and policies, and the diverse pensions and savings arrangements that fund later life (cf. Börsch-Supan et al., 2021). Might the dynamics underpinning these opt-in jobs extend beyond high-income countries to some middle-income or even low-income settings? There is evidence they do—Ranchhod (2006) assessed that social pensions enabled South African pensioners to take more flexible, part-time jobs, and Chen et al. (2021) observed similar tendencies among older adults who were working part-time as porters in a prosperous Chinese coastal area. Turning to the nature of opt-in jobs, a central topic is how the financial security offered by retirement income systems and other macro-level factors influence opt-in older workers’ motivations, priorities, and job quality (cf. Baumann et al., 2022; Wang et al., 2026). Answering these questions would contribute to broader discussions about the role of citizens’ incomes and other forms of social protection for labor market behavior and job quality.

Another area offering rich empirical insights is the study of processes underpinning older workers’ opt-in employment. Are opt-in older workers managing to shape their existing jobs to better fit their needs, or are they finding new jobs? Do the favorable working conditions of these jobs predominantly result from selection of already-advantaged workers into those jobs, or have older workers, more generally, managed to wend their way into better arrangements than before? Might the balance between these mechanisms vary in relation to organizational and macro-level factors? Qualitative research could trace the negotiations that give rise to these jobs, observing shifts in leverage between employer and employee, or client and self-employed worker. The presence of opt-in older workers may have consequences for the workforce as a whole: are these workers satisfying a need within the labor market and expanding economic output in the process (i.e., growing the economy) or might they be supplanting other workers seeking full-time jobs offering a living wage (i.e., extracting from a “fixed pie” view of the economy)? An open question is how far these workers are challenging life course norms: Might growing numbers of people working in the retirement years herald destandardization of normative constructions of the life course, influencing younger generations by challenging their conceptions of what is “on time” and what is “off time” (Justice et al., 2026; Settersten & Gannon, 2005)?

Lastly, key questions concern inequalities, and whether these jobs form part of cumulative advantage processes that deepen inequalities among older people (Cahill et al., 2024; Moen, 2016b): Does opt-in employment truly lift up older workers or does this work mainly compound advantages that have existed over decades of individuals’ working lives?

## Conclusion

It is our hope that the conceptual ideas presented in this paper will stimulate research that better captures the realities of work beyond standard pension and retirement ages in contemporary aging populations. We offer the insight that paid work takes on a distinctive character once retirement income systems help people to afford to retire. While many people leave the labor market at this point, those who continue voluntarily in paid work typically seek to maximize the intrinsic rewards of paid work while preserving their hard-won freedom over their time and energy. This paper evokes the distinctive experiences of these workers and proposes potential conditions and mechanisms that underlie the emergence of their jobs. For decades, these opt-in older workers have been hidden from view in plain sight. We suggest that serious study of these workers would offer novel insights for enduring questions across several fields, including the influence of social protection upon working lives, strategic interactions between employees and employers, and the changing priorities of older people themselves as they socially and economically age.

## Data Availability

The authors do not report data, and therefore the pre-registration and data availability requirements are not applicable.
